# The effects of telecommuting and family cohabiting situation on psychological distress in Japanese workers during the COVID‐19 pandemic: A cross‐sectional study

**DOI:** 10.1002/1348-9585.12391

**Published:** 2023-02-23

**Authors:** Atsuko Ikenouchi, Yoshihisa Fujino, Ryutaro Matsugaki, Kosuke Mafune, Hajime Ando, Tomohisa Nagata, Seiichiro Tateishi, Reiji Yoshimura, Mayumi Tsuji, for the CORoNaWork Project, Akira Ogami, Ayako Hino, Hisashi Eguchi, Keiji Muramatsu, Koji Mori, Makoto Okawara, Mami Kuwamura, Shinya Matsuda, Tomohiro Ishimaru, Yu Igarashi

**Affiliations:** ^1^ Medical Center for Dementia Hospital of the University of Occupational and Environmental Health, Japan Kitakyushu Japan; ^2^ Department of Psychiatry, School of Medicine University of Occupational and Environmental Health, Japan Kitakyushu Japan; ^3^ Department of Environmental Epidemiology Institute of Industrial Ecological Sciences, University of Occupational and Environmental Health, Japan Kitakyushu Japan; ^4^ Department of Preventive Medicine and Community Health, School of Medicine University of Occupational and Environmental Health, Japan Kitakyushu Japan; ^5^ Department of Mental Health Institute of Industrial Ecological Sciences, University of Occupational and Environmental Health, Japan Kitakyushu Japan; ^6^ Department of Work Systems and Health Institute of Industrial Ecological Sciences, University of Occupational and Environmental Health, Japan Kitakyushu Japan; ^7^ Department of Occupational Health Practice and Management Institute of Industrial Ecological Sciences, University of Occupational and Environmental Health, Japan Kitakyushu Japan; ^8^ Disaster Occupational Health Center Institute of Industrial Ecological Sciences, University of Occupational and Environmental Health, Japan Kitakyushu Japan; ^9^ Department of Environmental Health, School of Medicine University of Occupational and Environmental Health, Japan Kitakyushu Japan

**Keywords:** caregiving, cohabiting family, COVID‐19 pandemic, psychological distress, telecommuting

## Abstract

**Objective:**

This study aimed to determine whether telecommuting's impact on psychological distress differed depending on the status of workers' cohabiting family members during the COVID‐19 pandemic.

**Methods:**

We collected data from 33 302 workers in Japan through an Internet survey, and included 27 036 valid responses in the analysis. The survey included items on family cohabitation and telecommuting status during the COVID‐19 pandemic. We assessed workers' psychological distress using the Kessler 6.

**Results:**

The psychological distress odds ratios (ORs) were higher for participants who lived with family members requiring care (OR = 1.38, *P* < .001), and lower for participants living with preschool children (OR = 0.77, *P* < .001) or a spouse (OR = 0.80, *P* < .001). Furthermore, odds ratios were higher for participants who worked from home and lived with family members requiring care or preschool children (OR = 1.52, *P* = .002; OR = 1.28, *P* = .028). Stratified by the presence or absence of family members living with them, psychological distress was higher for telecommuters with family members requiring care, preschool children, or elementary school children.

**Conclusion:**

The association between telecommuting and psychological distress varies, depending on workers' living situation with family members.

## 1 INTRODUCTION

The COVID‐19 pandemic has progressed globally since the first case was reported in December 2019; as of July 2022, approximately 550 million cases have been confirmed, with more than 6 million deaths worldwide.^1^ Japan's first COVID‐19 infection was detected in January 2020; the number of infected people subsequently increased, especially in urban areas, and the infection rapidly spread to rural areas. The Japanese government declared a state of emergency in April 2020, asking people to stay home and close their workplaces. The broad movement restrictions implemented to suppress the pandemic reduced economic activity, which worsened working environments and corporate performance. This was followed by a period of infection spread, known as the second and third waves.^2^ This study was conducted during the third wave, when the number of infected people reached record daily highs.

Workplaces and commuting provide high‐risk opportunities for infection spread.^3^ Telecommuting was expected to decrease the risk of contracting COVID‐19^4^; therefore, telecommuting policies were promoted to control the pandemic through social distancing.^5^ Telecommuting implementation in Japan increased from 26.7% in March 2020 to 52.7% in May 2020.^6^ The pandemic increased telecommuting worldwide, and it continues to be widespread globally.^7^, ^8^

Telecommuting has differing impacts on workers' personal and professional lives. Positive effects include improved work–life balance, increased autonomy, more sleep time, increased productivity, and improved morale and job satisfaction^9^, ^10^ resulting from reduced commuting time and increased flexibility regarding where and when work is performed. Furthermore, studies have reported a positive relationship between telecommuting and organizational outcomes, including increased productivity, higher retention rates, stronger organizational commitment, and improved organizational performance.^11^

In contrast, telecommuting also has concerning effects, such as negative impacts on physical health, mental stress, and productivity. Telecommuting can involve overwork, tight deadlines, long work hours, and lack of rest periods, all of which are associated with fatigue, impaired mental and physical health, and reduced productivity.^10^ Moreover, long hours of continuous computer work at home can lead to musculoskeletal problems in the neck, shoulders, wrists, hands, and lower back owing to repetitive motion and unnatural posture.^10^ In addition, reduced supervisor and coworker support resulting from fewer opportunities for direct communication can lead to loneliness, isolation, anxiety, irritability, and depressed mood.^10^, ^12^ Telecommuting has also been associated with decreased sleep quality and mood disorders.^10^ Other telecommuting factors that affect productivity include the availability of suitable equipment and workspace, a stable Internet connection and power supply, support from leaders, roles and responsibilities in the company, and clarity of collaborators regarding the work.^13^

Cohabiting with family members may also affect telecommuting workers' health, stress, and productivity. Telecommuting blurs the boundary between work and home, which could lead to work–family conflicts, such as work interruptions to manage family demands and family life disruptions stemming from after‐hours work issues^14^; the difficulty of separating work and personal life when telecommuting may be a significant factor in psychological distress.^15^ For example, working women with children experience decreased happiness and work performance when caring for their children while working from home.^16^ It is important to support telecommuters and not force them to do double work (caring and working), because caring for dependents while working is difficult.^17^

Moreover, many workers who telecommuted during the COVID‐19 pandemic had to work from home without modifying their family environment, because situational demands, rather than personal choice, forced them to telecommute. The COVID‐19 pandemic closed or restricted the use of schools, daycare centers, and nursing homes; consequently, children and other family members requiring care were at home, and many workers needed to attend to these family members. Therefore, we hypothesized that workers living with children or family members who require care would experience increased psychological distress related to telecommuting. This study aimed to determine the impact of telecommuting on workers' psychological distress while living with family members needing care, preschool or elementary school children, or a spouse during the COVID‐19 pandemic.

## 2 METHODS

### 2.1 Study design and participants

We conducted an online survey for the Collaborative Online Research on the Novel‐Coronavirus and Work (CORoNaWork) project from December 22 to 26, 2020. The protocol for this cross‐sectional study was published previously in a peer‐reviewed journal.^18^ Participants included workers with full‐time jobs at the time of the study. The survey was conducted by Cross Marketing Inc., which has 4.7 million registered monitors. Invitations were sent via email to 605 381 individuals; 55 045 responded to the initial screening questions, and 33 302 met inclusion criteria (related to worker status, region, sex, and age) and completed the survey. However, 6051 respondents were excluded because of invalid responses or response errors. Exclusion criteria were designed to identify invalid responses, including extremely short response times (6 min), extremely low reported weight (<30 kg) and height (<140 cm), inconsistent responses to similar questions throughout the survey (e.g., marital status or region of residence), and incorrect responses to validity questions (e.g., “Please choose the third largest number from the following five numbers”). As reported in the published study protocol, some excluded group characteristics differed from those of the included group.^18^ In total, 27 036 valid responses were eligible for analysis. Participants were not compensated for participating in the survey.

This study was approved by the Ethics Committee of the University of Occupational and Environmental Health, Japan (reference No. R2‐079 and R3‐006). All participants provided informed consent after completing a form on the survey website. Data are not available owing to ethical restrictions and the need to preserve participants' anonymity.

### 2.2 Assessments

#### 2.2.1 Family cohabiting situation

We surveyed whether workers had preschool or elementary school children, family members requiring care, or a spouse living with them (e.g., “Do you have a preschooler living with you?”).

#### 2.2.2 Psychological distress

We used a validated Japanese version of the Kessler 6 (K6) scale to assess psychological distress,^19^, ^20^ and assigned a K6 score of 5 or higher as the psychological distress cut point.^20^

#### 2.2.3 Covariates

Covariates included age, sex, marital status, job type, annual equivalent household income, education, current smoking status, alcohol consumption, family cohabiting situation, telecommuting, and telecommuting preference.

### 2.3 Statistical analysis

We used a multilevel logistic model to estimate odds ratios (ORs) for psychological distress related to working from home while living with family members requiring care, preschool children, elementary school children, or a spouse, and to assess differences in participants' psychological distress based on the family cohabiting situation. We further estimated psychological distress ORs related to telecommuting, stratified by living with family members requiring care, preschool children, elementary school children, or a spouse.

The multivariate models were adjusted for age, sex, marital status, job type, annual equivalent household income, education, current smoking status, alcohol consumption, and telecommuting preference. All analyses were performed using Stata Statistical Software Release 17 (StataCorp LLC).

## 3 RESULTS

Table 1 presents participant characteristics. Of 27 036 participants, 2802 lived with their preschool children; of these, 512 (18.3%) worked from home and 1156 (41.3%) reported experiencing psychological distress. Furthermore, 1223 participants lived with family members requiring care; of these, 377 (30.8%) worked at home and 616 (50.4%) experienced psychological distress.

**TABLE 1:** Characteristics of participants who live with preschool children or family members requiring care.

Factors	Without preschool children	With preschool children	Without family members requiring care	With family members requiring care
*N*	24 234	2802	25 813	1223
Age, median (interquartile range)	49.0 (42.0, 56.0)	37.0 (32.0, 43.0)	48.0 (39.0, 55.0)	52.0 (43.0, 58.0)

	*N* (%)	*N* (%)	*N* (%)	*N* (%)
Sex, male	12 722 (52.5%)	1092 (39.0%)	13 154 (51.0%)	660 (54.0%)
Marital status, married	12 429 (51.3%)	2600 (92.8%)	14 451 (56.0%)	578 (47.3%)
Job type				
Mainly desk work	12 190 (50.3%)	1278 (45.6%)	12 854 (49.8%)	614 (50.2%)
Jobs mainly involving interpersonal communication	6074 (25.1%)	853 (30.4%)	6611 (25.6%)	316 (25.8%)
Mainly labor	5970 (24.6%)	671 (23.9%)	6348 (24.6%)	293 (24.0%)
Annual equivalent household income				
0.5–2.49 million yen	5137 (21.2%)	573 (20.4%)	5324 (20.6%)	386 (31.6%)
2.5–3.74 million yen	6624 (27.3%)	926 (33.0%)	7233 (28.0%)	317 (25.9%)
3.75–4.89 million yen	5763 (23.8%)	862 (30.8%)	6289 (24.4%)	336 (27.5%)
More than 4.90 million yen	6710 (27.7%)	441 (15.7%)	6967 (27.0%)	184 (15.0%)
Education				
Junior high school	338 (1.4%)	30 (1.1%)	352 (1.4%)	16 (1.3%)
High school	6391 (26.4%)	562 (20.1%)	6640 (25.7%)	313 (25.6%)
Vocational school/ College/University/ Graduate school	17 505 (72.2%)	2210 (78.9%)	18 821 (72.9%)	894 (73.1%)
Current smoking status	6464 (26.7%)	540 (19.3%)	6615 (25.6%)	389 (31.8%)
Alcohol consumption				
6–7 days per week	5236 (21.6%)	438 (15.6%)	5386 (20.9%)	288 (23.5%)
4–5 days per week	1882 (7.8%)	195 (7.0%)	1946 (7.5%)	131 (10.7%)
2–3 days per week	2976 (12.3%)	290 (10.3%)	3102 (12.0%)	164 (13.4%)
Less than 1 day per week	4092 (16.9%)	455 (16.2%)	4367 (16.9%)	180 (14.7%)
Almost none	10 048 (41.5%)	1424 (50.8%)	11 012 (42.7%)	460 (37.6%)
Family cohabiting situation				
With family members requiring care	1097 (4.5%)	126 (4.5%)	0 (0.0%)	1223 (100.0%)
With preschool children	0 (0.0%)	2802 (100.0%)	2676 (10.4%)	126 (10.3%)
With elementary school children	1888 (7.8%)	820 (29.3%)	2569 (10.0%)	139 (11.4%)
With spouse	11 901 (49.1%)	2553 (91.1%)	13 904 (53.9%)	550 (45.0%)
Telecommuting	5248 (21.7%)	512 (18.3%)	5383 (20.9%)	377 (30.8%)
Telecommuting preference	7470 (30.8%)	1128 (40.3%)	8153 (31.6%)	445 (36.4%)
Psychological distress (K6 ≥ 5)	9661 (39.9%)	1156 (41.3%)	10 201 (39.5%)	616 (50.4%)

Table 2 shows psychological distress ORs related to participants' family cohabiting situation and telecommuting status, as estimated by the logistic model. Psychological distress ORs of workers who lived with family members requiring care were significantly higher than those who did not (OR = 1.45, 95% confidence interval [CI] 1.26–1.68, *P* < .001). A similar result was obtained in the multivariate analysis (OR = 1.38, 95% CI 1.19–1.59, *P* < .001). In contrast, the psychological distress ORs for workers who lived with preschool children or a spouse were significantly lower than those of workers who did not (OR = 0.81, 95% CI 0.73–0.89, *P* < .001 and OR = 0.74, 95% CI 0.70–0.79, *P* < .001, respectively). The same was true in the multivariate analysis (OR = 0.77, 95% CI 0.69–0.85, *P* < .001 and OR = 0.80, 95% CI 0.75–0.86, *P* < .001, respectively). In the multivariate analysis, psychological distress ORs were higher for workers who telecommuted than for those who did not (OR = 1.18, 95% CI 1.04–1.34, *P* = .009). Psychological distress ORs were also higher for those who worked at home and lived with family members requiring care, or with preschool children (OR = 1.52, 95% CI 1.18–1.97, *P* = .001, and OR = 1.27, 95% CI 1.02–1.58, *P* = .031, respectively). The multivariate analysis showed a similar pattern (OR = 1.52, 95% CI 1.17–1.97, *P* = .002; OR = 1.28, 95% CI 1.03–1.59, *P* = .028).

**TABLE 2:** Association between family cohabiting situation, telecommuting, and psychological distress.

	Age–sex adjusted				Multivariate^a^			
	OR	95% CI		*P*‐value	OR	95% CI		*P*‐value
Family cohabiting situation								
Without family members requiring care	Reference				Reference			
With family members requiring care	1.45	1.26	1.68	<.001	1.38	1.19	1.59	<.001
Without preschool children	Reference				Reference			
With preschool children	0.81	0.73	0.89	<.001	0.77	0.69	0.85	<.001
Without elementary school children	Reference				Reference			
With elementary school children	1.02	0.93	1.13	.641	0.95	0.86	1.04	.271
Without spouse	Reference				Reference			
With spouse	0.74	0.70	0.79	<.001	0.80	0.75	0.86	<.001
Without telecommuting	Reference				Reference			
With telecommuting	1.05	0.93	1.19	.413	1.18	1.04	1.34	.009
Interaction between family cohabiting situation and telecommuting								
Telecommuting × with family members requiring care	1.52	1.18	1.97	.001	1.52	1.17	1.97	.002
Telecommuting × with preschool children	1.27	1.02	1.58	.031	1.28	1.03	1.59	.028
Telecommuting × with elementary school children	1.18	0.95	1.45	.132	1.19	0.96	1.47	.109
Telecommuting × with spouse	0.93	0.82	1.06	.280	0.91	0.80	1.03	.140

Abbreviations: CI, confidence interval; OR, odds ratio.

^a^ Adjusted for age, sex, marital status, job type, annual equivalent household income, education, current smoking status, alcohol consumption, and telecommuting preference.

Table 3 shows telecommuting workers' psychological distress ORs stratified by whether they lived with a family member requiring care, preschool children, elementary school children, or a spouse. Workers who lived with family members requiring care, preschool children, or elementary school children had higher psychological distress ORs than those who did not. The multivariate analysis showed a similar trend. Psychological distress ORs among telecommuting workers did not differ according to whether they lived with a spouse, and the multivariate analysis showed a similar trend.

**TABLE 3:** Association between family cohabiting situation and psychological distress during telecommuting.

	Age–sex adjusted				Multivariate^a^			
	OR	95% CI		*P*‐value	OR	95% CI		*P*‐value
Stratified by existence of family members requiring care								
Telecommuting without a family member requiring care	0.99	0.93	1.06	.791	1.09	1.02	1.17	.015
Telecommuting with a family member requiring care	1.52	1.18	1.96	.001	1.53	1.15	2.04	.004
Stratified by existence of preschool children								
Telecommuting without preschool children	1.02	0.95	1.08	.629	1.10	1.02	1.18	.009
Telecommuting with preschool children	1.23	1.01	1.50	.042	1.26	1.02	1.56	.031
Stratified by existence of elementary school children								
Telecommuting without elementary school children	1.01	0.95	1.08	.791	1.09	1.02	1.17	.012
Telecommuting with elementary school children	1.24	1.02	1.50	.028	1.24	1.01	1.52	.039
Stratified by existence of spouse								
Telecommuting without spouse	1.03	0.95	1.13	.452	1.11	1.01	1.23	.029
Telecommuting with spouse	1.02	0.94	1.11	.663	1.11	1.01	1.21	.027

*Note*: Reference for comparison is without telecommuting. Abbreviations: CI, confidence interval; OR, odds ratio.

^a^ Adjusted for age, sex, marital status, job type, annual equivalent household income, education, current smoking status, alcohol consumption, and telecommuting preference.

## 4 DISCUSSION

This study found that workers who telecommuted during the COVID‐19 pandemic experienced higher levels of psychological distress than those who did not. The results also showed a significant interaction between workers' family cohabiting situation and telecommuting, where the degree of psychological distress varied, depending on whether the workers lived with family members requiring care or with preschool children. Telecommuting workers who resided with family members requiring care or with children reported higher psychological distress than those who did not. These results reveal psychological distress associated with telecommuting from the perspective of workers' family cohabiting situation.

The relationship between telecommuting and psychological distress may vary by family cohabiting situation because of conflicts in family relationships. Telecommuting can increase the conflict between work and family because it is difficult to establish boundaries between professional and personal lives. The increased flexibility offered by telecommuting may also result in increased stress related to family responsibilities, difficulty separating work and home, and family conflicts.^10^, ^14^, ^21^ Telecommuting workers often need to interrupt work activities to deal with family demands and frequently continue to participate in work roles and activities after work hours.^14^ These situations may involve “family–work conflicts” when family responsibilities affect work activities and “work–family conflicts” when work obligations affect family care.^22^ The blurred boundaries can lead to longer work hours, less spare time, and burnout.^23^

This study showed that living with a family member in need of care prompted an association between telecommuting and psychological distress. Soubelet‐Fagoaga et al. showed that workers with caregiving responsibilities—especially women who telework—experience significant difficulties with balancing work and home life. The inability to balance care and telework creates particularly stressful situations.^17^ Caregivers who telecommute have significantly higher stress levels and significantly lower satisfaction with telecommuting than non‐caregivers, and nearly 30% of caregivers cite their home environment as the biggest challenge to telecommuting.^24^

This study also found that preschool children's presence strengthens the association between telecommuting and psychological distress. Before the COVID‐19 pandemic, telecommuting workers with children scored higher on satisfaction and family well‐being than those without children; however, during the COVID‐19 pandemic, telecommuting workers with children were not significantly more satisfied with their telecommuting.^9^, ^25^ Reports showed that, during the COVID‐19 lockdown, telecommuting resulted in increased domestic difficulties, such as school closure, childcare, and child health responsibilities; increased housework; blurred boundaries between home and work; and longer working hours. Mental health issues emerged, including anxiety, depression, fatigue, burnout, and lack of an outlet for stress.^26^ Furthermore, approximately half of telecommuting women with children under age 18 reported that their children's age affected their performance.^27^

Telecommuting challenges associated with children and family members requiring care were further emphasized during the COVID‐19 pandemic period, when day‐care centers, nursing homes, and elderly day services were closed. This may have impaired workers' control over their jobs, causing distress related to caring for family members or preschool children who lived with them.^28^, ^29^ In the absence of an appropriate workspace, workers and children must adapt to sharing the same work and childcare environment; connectivity issues and lack of focus can increase the challenge.^26^ Moreover, middle‐aged and older adults who work from home full time while providing emotional and financial support to their minor children, may simultaneously be providing physical, financial, and legal support to their older parents. This can lead to high stress and long work hours as they juggle the demands of caring for both their children and their parents.^30^ Although these workers could previously refresh themselves by going to their workplace, telecommuting made this difficult; many were then forced to simultaneously work and care for family members at home. The burden of family caregiving has been shown to cause psychological distress and loneliness^31^, ^32^; those who live with family members who require care are even more likely to experience distress related to caregiving.^33^

Our findings indicate that telecommuting workers who live with family members requiring care or preschool children are likely to experience psychological distress. Several studies have evaluated the impact of telecommuting on mental health, but the conclusions have been inconsistent.^12^, ^34^, ^35^, ^36^ To the best of our knowledge, this is the first study to examine the impact of telecommuting by considering the family's cohabitation status. Studies conducted before the COVID‐19 pandemic showed that telecommuting's flexibility gives workers greater control over their lives and improves life–work balance^37^, ^38^; therefore, regular telecommuters showed fewer work–family conflicts and fewer stressors.^21^, ^39^ In addition, time spent with family was believed to contribute to well‐being through social integration, increased self‐importance, and easier access to social support.^40^ There are no previous reports of telecommuting during other pandemics, such as the severe acute respiratory syndrome (SARS) or the Middle East respiratory syndrome (MERS) pandemics; therefore, we could not make direct comparisons. However, there may be similarities among respiratory infections that lead to global pandemics. COVID‐19 emerged unexpectedly, leaving companies and workers inadequately prepared for telecommuting, which may have contributed to our findings.

Most companies do not have telecommuting work rules, and some have no online access to company resources. Many workers were forced to telecommute because they needed to care for family members who lived with them, but many did not have space to perform their work or lived in an environment that was not optimal for work. Therefore, it is important to consider the work environment and family situation when introducing telecommuting. Furthermore, it is necessary to understand the psychological distress workers face when working from home and provide them with the option of working in the office to obtain psychological support. Companies should also consider measures to reduce distress experienced by workers who live with family members requiring care when working from home. It can be difficult to separate work and personal lives during telecommuting; therefore, to separate work and home in relation to time and space, it may be necessary to hang up the work phone at the end of the day, avoid receiving calls or calling outside of work hours, and to secure a home office. Telecommuting may also affect workers' spare time; therefore, it may be helpful to establish routines for regular eating, sleeping, and exercise, to prevent overwork and mental anguish resulting from less resting time. After implementing infection control measures, continuing business operations that provide social service resources, such as nursing care and childcare, would also help support telecommuting workers living with someone who needs care.

This study has several limitations. First, because this was an Internet survey, selection bias was inevitable. To reduce bias as much as possible, we selected a diverse sample of participants in terms of sex, occupation, and region, based on COVID‐19 incidence. Second, because of the cross‐sectional design, unmeasured confounders and causal relationships could not be clarified. Third, the study was conducted during the COVID‐19 pandemic, and it is unclear how the pandemic and associated changes in daily life and occupational environment affected the results. Fourth, common method bias was unavoidable because of the large number of standard questionnaire items used in Internet surveys. However, the K6 self‐report outcome measure has a variance explained by Harman's single factor test of 25%, which is below 50%; therefore, the effect of common method bias was assumed to be small. Fifth, the telecommuting designation validity and reliability are unknown because telecommuting status was examined with a simple question.

## 5 CONCLUSIONS

Associations between telecommuting and psychological distress depend on workers' family living situations. During the COVID‐19 pandemic, workers living with family members requiring care experienced higher psychological distress, while those living with a spouse or preschool children had lower psychological distress. Telecommuting was associated with increased psychological distress among those living with family members requiring care or young children. This may be partly because telecommuting was introduced in an emergency without consideration of workers' family cohabiting situations. In telecommuting during an emergency, such as the COVID‐19 pandemic, it is important to provide psychological and social support that considers workers' situations regarding family members living with them.

## AUTHOR CONTRIBUTIONS

Atsuko Ikenouchi: Conceptualization, investigation, data curation, and writing—original draft preparation. Yoshihisa Fujino: Conceptualization, methodology, software, formal analysis, investigation, data curation, writing—original draft preparation, project administration, and funding acquisition. Ryutaro Matsugaki: Investigation, data curation, and writing—review and editing. Kosuke Mafune: Investigation, data curation, and writing—review and editing. Hajime Ando: Investigation, data curation, and writing—review and editing. Tomohisa Nagata: Investigation, data curation, writing—review and editing, and funding acquisition. Seiichiro Tateishi: Investigation, data curation, writing—review and editing, and funding acquisition. Reiji Yoshimura: Investigation, data curation, and writing—original draft preparation. Mayumi Tsuji: Investigation, data curation, writing—review and editing, and funding acquisition.

## FUNDING INFORMATION

This study was supported and partly funded by a research grant from the University of Occupational and Environmental Health, Japan (no grant number); the Japanese Ministry of Health, Labour and Welfare (H30‐josei‐ippan‐002, H30‐roudou‐ippan‐007, 19JA1004, 20JA1006, 210301–1, and 20HB1004); Anshin Zaidan (no grant number); the Collabo‐Health Study Group (no grant number); Hitachi Systems, Ltd. (no grant number); and scholarship donations from Chugai Pharmaceutical Co., Ltd. (no grant number). The funders were not involved in the study design, collection, analysis, interpretation of data, writing of this article, or the decision to submit it for publication.

## CONFLICT OF INTEREST STATEMENT

The authors declare no conflict of interests for this article.

## DISCLOSURE

This study was approved by the Ethics Committee of the University of Occupational and Environmental Health, Japan (reference No. R2‐079 and R3‐006). All participants provided informed consent after completing a form on the survey website.

## Data Availability

Data are not available owing to ethical restrictions and the need to preserve participants' anonymity.

## References

[1] COVID‐19 Data Explorer . Our world data. Retrieved from https://ourworldindata.org/explorers/coronavirus‐data‐explorer; 2022.

[2] Novel Coronavirus (COVID‐19) . The Japan Institute for Labour Policy and Training. Retrieved from https://www.jil.go.jp/english/special/covid‐19/index.html; 2022.

[3] Hu M , Lin H , Wang J , et al. Risk of coronavirus disease 2019 transmission in train passengers: an epidemiological and modeling study. Clin Infect Dis. 2021;72(4):604‐610. doi: 10.1093/cid/ciaa1057 32726405 PMC7454391

[4] Galmiche S , Charmet T , Schaeffer L , et al. Exposures associated with SARS‐CoV‐2 infection in France: a nationwide online case‐control study. Lancet Reg Health. 2021;7: 100148. doi: 10.1016/j.lanepe.2021.100148 PMC8183123 34124709

[5] Anderson RM , Heesterbeek H , Klinkenberg D , Hollingsworth TD . How will country‐based mitigation measures influence the course of the COVID‐19 epidemic? Lancet. 2020;395(10228):931‐934. doi: 10.1016/S0140-6736(20)30567-5 32164834 PMC7158572

[6] Sasaki N , Imamura K , Kataoka M , et al. COVID‐19 measurements at the workplace in various industries and company sizes: a 2‐month follow‐up cohort study of full‐time employees in Japan. Environ Occup Health Pract. 2021;3. doi: 10.1539/eohp.2020-0017-OA

[7] Ahrendt D , Cabrita J , Clerici E , et al. Living, working and COVID‐19. Retrieved from https://www.eurofound.europa.eu/publications/report/2020/living‐working‐and‐covid‐19; 2020.

[8] Dingel JI , Neiman B . How many jobs can be done at home? J Public Econ. 2020;189: 104235. doi: 10.1016/j.jpubeco.2020.104235 32834177 PMC7346841

[9] Sousa‐Uva M , Sousa‐Uva A , Sampayo MME , Serranheira F . Telework during the COVID‐19 epidemic in Portugal and determinants of job satisfaction: a cross‐sectional study. BMC Public Health. 2021;21(1):2217. doi: 10.1186/s12889-021-12295-2 34865641 PMC8645416

[10] Tavares AI . Telework and health effects review. Int J Healthc. 2017;3(2):30. doi:10.5430/ijh.v3n2p30

[11] Martin BH , MacDonnell R . Is telework effective for organizations?: a meta‐analysis of empirical research on perceptions of telework and organizational outcomes. Manag Res Rev. 2012;35(7):602‐616. doi: 10.1108/01409171211238820

[12] Mann S , Holdsworth L . The psychological impact of teleworking: stress, emotions and health. New Technol Work Employ. 2003;18(3):196‐211. doi: 10.1111/1468-005X.00121

[13] Rodrigues EA , Rampasso IS , Serafim MP , Filho WL , Anholon R . Productivity analysis in work from home modality: an exploratory study considering an emerging country scenario in the COVID‐19 context. Work. 2022;72(1):39‐48. doi: 10.3233/WOR-211212 35431219

[14] Delanoeije J , Verbruggen M , Germeys L . Boundary role transitions: a day‐to‐day approach to explain the effects of home‐based telework on work‐to‐home conflict and home‐to‐work conflict. Hum Relat. 2019;72(12):1843‐1868. doi: 10.1177/0018726718823071

[15] Nagata T , Ito D , Nagata M , et al. Anticipated health effects and proposed countermeasures following the immediate introduction of telework in response to the spread of COVID‐19: the findings of a rapid health impact assessment in Japan. J Occup Health. 2021;63(1): e12198. doi: 10.1002/1348-9585.12198 33527667 PMC7851629

[16] Shockley KM , Clark MA , Dodd H , King EB . Work‐family strategies during COVID‐19: examining gender dynamics among dual‐earner couples with young children. J Appl Psychol. 2021;106(1):15‐28. doi: 10.1037/apl0000857 33151705

[17] Soubelet‐Fagoaga I , Arnoso‐Martínez M , Guerendiain‐Gabás I , Martínez‐Moreno E , Ortiz G . (tele)work and care during lockdown: labour and socio‐familial restructuring in times of COVID‐19. Int J Environ Res Public Health. 2021;18(22):12087. doi: 10.3390/ijerph182212087 34831843 PMC8620492

[18] Fujino Y , Ishimaru T , Eguchi H , et al. Protocol for a nationwide internet‐based health survey of workers during the COVID‐19 pandemic in 2020. J UOEH. 2021;43(2):217‐225. doi: 10.7888/juoeh.43.217 34092766

[19] Kessler RC , Andrews G , Colpe LJ , et al. Short screening scales to monitor population prevalences and trends in non‐specific psychological distress. Psychol Med. 2002;32(6):959‐976. doi: 10.1017/s0033291702006074 12214795

[20] Furukawa TA , Kawakami N , Saitoh M , et al. The performance of the Japanese version of the K6 and K10 in the world mental health survey Japan. Int J Methods Psychiatr Res. 2008;17(3):152‐158. doi: 10.1002/mpr.257 18763695 PMC6878390

[21] Golden TD , Veiga JF , Simsek Z . Telecommuting's differential impact on work‐family conflict: is there no place like home? J Appl Psychol. 2006;91(6):1340‐1350. doi: 10.1037/0021-9010.91.6.1340 17100488

[22] Netemeyer RG , Boles JS , McMurrian R . Development and validation of work–family conflict and family–work conflict scales. J Appl Psychol. 1996;81(4):400‐410. doi: 10.1037/0021-9010.81.4.400

[23] Allvin M , Aronsson G , Hagström T , Johansson G , Lundberg U , eds. Work without Boundaries: Psychological Perspectives on the New Working Life. John Wiley & Sons, Ltd; 2011.

[24] Danker TN , Yap HL , Zalzuli AD , Ho HF , Ang J . Surviving work from home: observations from Singapore. J Police Crim Psychol. 2022;37(2):407‐422. doi: 10.1007/s11896-021-09461-y 34334936 PMC8300063

[25] Mokhtarian PL , Bagley MN , Salomon I . The impact of gender, occupation, and presence of children on telecommuting motivations and constraints. J Am Soc Inf Sci. 1998;49(12):1115‐1134. doi: 10.1002/(SICI)1097-4571(1998)49:12<1115::AID-ASI7>3.0.CO;2-Y

[26] Bezak E , Carson‐Chahhoud KV , Marcu LG , et al. The biggest challenges resulting from the COVID‐19 pandemic on gender‐related work from home in biomedical fields—world‐wide qualitative survey analysis. Int J Environ Res Public Health. 2022;19(5):3109. doi: 10.3390/ijerph19053109 35270801 PMC8910706

[27] Costa C , Teodoro M , Mento C , et al. Work performance, mood and sleep alterations in home office workers during the COVID‐19 pandemic. Int J Environ Res Public Health. 2022;19(4): 1990. doi: 10.3390/ijerph19041990 35206177 PMC8871883

[28] Shinomiya Y , Yoshizaki A , Murata E , Fujisawa T , Taniike M , Mohri I . Sleep and the general behavior of infants and parents during the closure of schools as a result of the COVID‐19 pandemic: comparison with 2019 data. Children. 2021;8(2): 168. doi: 10.3390/children8020168 33671816 PMC7926370

[29] Zarit SH , Kim K , Femia EE , Almeida DM , Klein LC . The effects of adult day services on family caregivers' daily stress, affect, and health: outcomes from the daily stress and health (DaSH) study. Gerontologist. 2014;54(4):570‐579. doi: 10.1093/geront/gnt045 23690056 PMC4155447

[30] Sugestyo Putro S , Riyanto S . How Asian sandwich generation managing stress in telecommuting during Covid‐19 pandemic. IJSRE. 2020;3(3):485‐492. Retrieved from http://www.ijsred.com/volume3/issue3/IJSRED‐V3I3P64.pdf

[31] Bramboeck V , Moeller K , Marksteiner J , Kaufmann L . Loneliness and burden perceived by family caregivers of patients with Alzheimer disease. Am J Alzheimers Dis Other Demen. 2020;35: 1533317520917788. doi: 10.1177/1533317520917788 32281389 PMC10624003

[32] Fujii R , Konno Y , Tateishi S , et al. Association between time spent with family and loneliness among Japanese workers during the COVID‐19 pandemic: a cross‐sectional study. Front Psych. 2021;12: 786400. doi: 10.3389/fpsyt.2021.786400 PMC8692760 34955931

[33] Schulz R , Beach SR , Czaja SJ , Martire LM , Monin JK . Family caregiving for older adults. Annu Rev Psycho. 2020;71(1):635‐659. doi: 10.1146/annurev-psych-010419-050754 PMC7291827 31905111

[34] Chow JSF , Palamidas D , Marshall S , Loomes W , Snook S , Leon R . Teleworking from home experiences during the COVID‐19 pandemic among public health workers (TelEx COVID‐19 study). BMC Public Health. 2022;22(1):674. doi: 10.1186/s12889-022-13031-0 35392859 PMC8988115

[35] Shimura A , Yokoi K , Ishibashi Y , Akatsuka Y , Inoue T . Remote work decreases psychological and physical stress responses, but full‐remote work increases presenteeism. Front Psychol. 2021;12: 730969. doi: 10.3389/fpsyg.2021.730969 34659039 PMC8514617

[36] Trógolo MA , Moretti LS , Medrano LA . A nationwide cross‐sectional study of workers' mental health during the COVID‐19 pandemic: impact of changes in working conditions, financial hardships, psychological detachment from work and work‐family interface. BMC Psychol. 2022;10(1):73. doi: 10.1186/s40359-022-00783-y 35303966 PMC8931581

[37] Beauregard TA , Basile KA , Canonico E . Telework. In: Landers RN , ed. The Cambridge Handbook of Technology and Employee Behavior. Cambridge University Press; 2019:511‐543.

[38] Bloom N , Liang J , Roberts J , Ying ZJ . Does working from home work? Evidence from a Chinese experiment. Q J Econ. 2015;130(1):165‐218. doi: 10.1093/qje/qju032

[39] Hill EJ , Erickson JJ , Holmes EK , Ferris M . Workplace flexibility, work hours, and work‐life conflict: finding an extra day or two. J Fam Psychol. 2010;24(3):349‐358. doi: 10.1037/a0019282 20545408

[40] Ellicott S , Umberson D . Recent demographic trends in the US and implications for well‐being. In: Scott J , Richards TJ , eds. The Blackwell Companion to the Sociology of Families. Blackwell Publishing Ltd; 2004:34‐53.

